# Liver toxicity from vitamin A

**DOI:** 10.1002/jgh3.12201

**Published:** 2019-05-30

**Authors:** Rosanna Fox, Nigel Stace, Karen Wood, Claire French

**Affiliations:** ^1^ Department of Gastroenterology & Hepatology Wellington Regional Hospital Wellington New Zealand

**Keywords:** drug‐induced liver injury, hepatic inflammation, hypervitaminosis A

## Abstract

Hepatic toxicity secondary to hypervitaminosis A is extremely rare. We report the case of a 27‐year‐old Caucasian female who presented for an investigation of abdominal pain, cholestatic liver function tests, and abnormal computerized tomography findings. She had been prescribed isotretinoin for her acne and had subsequently purchased vitamin A online, which she consumed daily for over 18 months.

## Graphical abstract

This is a case report of a young female presenting with abnormal liver function and ascites secondary to hypervitaminosis A. The patient was ordering vitamin A online to self‐treat her acne.

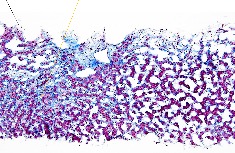

## Introduction

A 27‐year‐old Caucasian female presented to the emergency department with a 2‐year history of postprandial abdominal pain. She reported intermittent bloating and a 10‐kg weight gain over 12 months and complained that her abdomen was distended and tense. Her body mass index (BMI) was 21.8 kg/m^2^.

## Case Report

Investigations showed a normal full blood count (platelets 350 × 10^9^/L) but cholestatic liver tests, with the following measurements: bilirubin 15 μg/dL, alkaline phosphatase (ALP) 208 U/L, gamma‐glutamyltransferase (GGT) 237 U/L, alanine aminotransaminase (ALT) 15 U/L, aspartate aminotransaminase (AST) 28 U/L, albumin 41 g/L, international normalized ratio (INR) 1.2, sodium 139 mmol/L, and creatinine 90 μmol/L. The Model for End‐Stage Liver Disease (MELD) score was 9. Blood tests were normal or negative for viral, metabolic, and immune causes of chronic liver disease, including Wilson's disease. A thrombophilia screen was negative. Earlier, a community abdominal ultrasound was normal, but a computerized tomography (CT) scan showed “a heterogeneous‐appearing enlarged liver with especially caudate lobe hypertrophy …Compression of IVC and left hepatic vein…and moderate volume ascites without splenomegaly.”

Her past medical history included acne, migraines, and bilateral ureteric reimplantation at 6 years of age. She was taking a combined oral contraceptive, vitamin C, and vitamin A. Historically, she had taken two 5‐month courses of isotretinoin 20 mg daily from August 2013 to January 2014 and again from June to November 2014. Whilst helpful for her acne, there were significant side effects, including dry skin and eyes and oral and anorectal fissuring, so later, the patient began self‐treatment with vitamin A purchased online. Each capsule contained retinyl palmitate of 10 000 IU (or 3 mg retinol), and the bottle was labeled “Dietary Supplement.” The recommended daily intake for women is 500 μg/day of retinol.^1^ This supplement was taken daily for a total period of about 18 months from January 2015. It was not being taken at the time of presentation in October 2017.

On examination, there was mild palmar erythema and several small spider naevi on the anterior chest. The abdomen was distended and slightly firm, and there was percussion tenderness over the lower right ribs. There was no palpable organomegaly or peripheral edema.

Doppler ultrasound showed normal flow and caliber in the three hepatic veins and in the portal vein.

The serum minus the ascites albumin gradient was 21 g/L, indicating a transudate.

**Figure 1:**
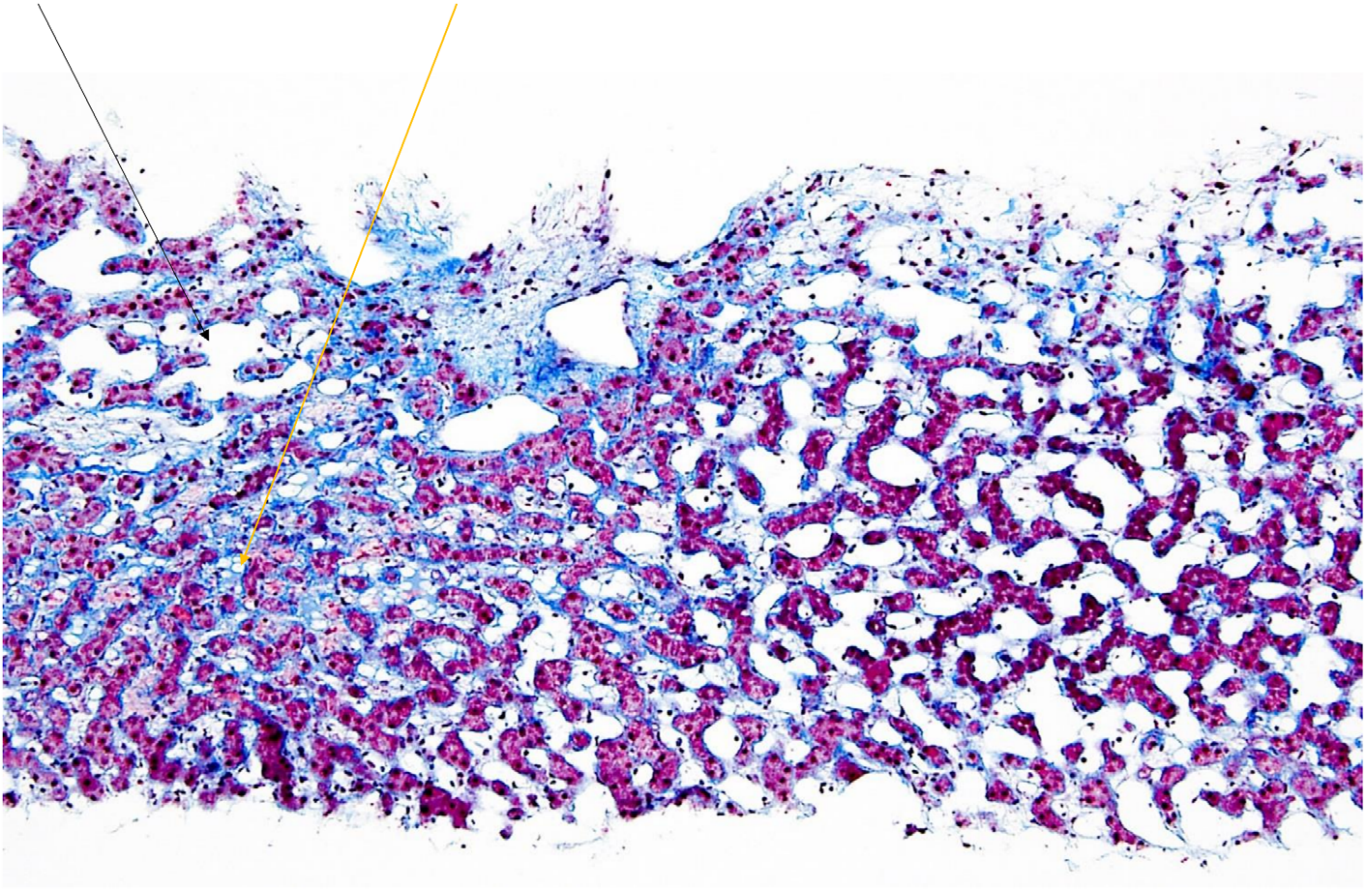
A liver biopsy at 20× magnification stained with trichrome showing extensive pericentral and perisinusoidal fibrosis (blue collagen deposition), with dilation of the sinusoids (black arrow) associated with hepatocyte atrophy. The stellate cells are hypertrophied and contain fat vacuoles (yellow arrow). This pattern of fibrosis, perisinusoidal dilatation, and hypertrophied stellate cells is consistent with hypervitaminosis A.

Liver biopsy as shown in Figure 1 demonstrated parenchymal abnormality around the central veins, with pericentral fibrosis, central vein thrombosis, and prominent sinusoidal fibrosis. The hepatic sinusoids contained enlarged, clear stellate cells, giving a “bubbly appearance.” Connective tissue stains showed marked collagen deposition in the pericentral and perisinusoidal areas. The stellate cells also contain fat vacuoles, which is consistent with hypervitaminosis A.

This patient is currently clinically well and will be followed up in outpatient clinic. She has stopped all oral vitamin supplements.

## Discussion

Vitamin A is a fat‐soluble vitamin involved in the maintenance of normal immune, visual, and reproductive function.^1^ Despite hypervitaminosis A being a well‐documented cause of liver cirrhosis,^2^, ^3^, ^4^ there are very few case studies in the recent literature.^5^, ^6^, ^7^ Indeed, combined data from the Australian Database of Adverse Event Notifications and the New Zealand Centre for Adverse Reactions Monitoring report only three individual cases of vitamin A‐/retinol‐associated hepatobiliary disorders in the last 50 years.^8^, ^9^

An increasing number of patients are self‐treating with unregulated medications purchased over‐the‐counter or online^10^; it is important that the risks of seemingly innocuous vitamins are highlighted to both patients and medical professionals. This case study demonstrates the risk of hypervitaminosis A and the possible need for restrictions on sales of unprescribed vitamin A. It is important that those prescribing vitamin A‐containing medications are aware of these risks and monitor patients according to prescribing recommendations. A thorough drug history should be taken for all patients who are being investigated for abnormal liver function, and vitamin A at any dose should be considered a possible cause for deranged liver function.^11^, ^12^
